# Potential Biomarkers of the Earliest Clinical Stages of Parkinson's Disease

**DOI:** 10.1155/2015/294396

**Published:** 2015-09-21

**Authors:** Anelya Kh. Alieva, Elena V. Filatova, Aleksey V. Karabanov, Sergey N. Illarioshkin, Petr A. Slominsky, Maria I. Shadrina

**Affiliations:** ^1^Institute of Molecular Genetics, Russian Academy of Sciences, Moscow 123182, Russia; ^2^Research Centre of Neurology, Moscow 125367, Russia

## Abstract

Parkinson's disease (PD) is a widespread neurodegenerative disorder. Despite the intensive studies of this pathology, in general, the picture of the etiopathogenesis has still not been clarified fully. To understand better the mechanisms underlying the pathogenesis of PD, we analyzed the expression of 10 genes in the peripheral blood of treated and untreated patients with PD. 35 untreated patients with PD and 12 treated patients with Parkinson's disease (Hoehn and Yahr scores 1-2) were studied. An analysis of the mRNA levels of *ATP13A2*, *PARK2*, *PARK7*, *PINK1*, *LRRK2*, *SNCA*, *ALDH1A1*, *PDHB*, *PPARGC1A*, and *ZNF746* genes in the peripheral blood of patients was carried out using reverse transcription followed by real-time PCR. A statistically significant and specific increase by more than 1.5-fold in the expression of the *ATP13A2*, *PARK7*, and *ZNF746* genes was observed in patients with PD. Based on these results, it can be suggested that the upregulation of the mRNA levels of *ATP13A2*, *PARK7*, and *ZNF746* in untreated patients in the earliest clinical stages can also be observed in the preclinical stages of PD, and that these genes can be considered as potential biomarkers of the preclinical stage of PD.

## 1. Introduction

Parkinson's disease (PD) is a widespread neurodegenerative disorder that is characterized by the degeneration of dopaminergic (DA) neurons of the midbrain [1]. Resting tremor, bradykinesia, rigidity, and postural instability are the main clinical manifestations of this disease. One of the features of PD is that its basic clinical signs appear in the late stages of the development of the neurodegenerative processes in the pars compacta of the substantia nigra, when about 60% of the DA neurons of the substantia nigra have died and dopamine levels in the striatum have decreased by 80% [2, 3]. From a genetic point of view, PD is a heterogeneous pathology. In most cases, PD is sporadic. The proportion of monogenic cases varies from 10% to 15% in different populations [4]. Intensive studies carried out over the past 15 years have revealed the involvement of a large number of different loci and genes in the development of PD [5]. Despite these advances, the molecular genetic mechanisms that trigger the development of PD remain unknown, and, in general, the picture of the etiopathogenesis of PD has still not been clarified fully. There is no doubt that mutations and single-nucleotide polymorphisms associated with the development of PD play an important role in the pathogenesis of this disease. However, in addition to violations of the structure of DNA, changes at other cellular levels, including the transcriptome level, may also play a significant role in the pathogenesis of PD.

To date, several studies of transcriptome alterations have been carried out in the postmortem brains of patients with PD. The patients analyzed in those studies were in the last and most severe stages of the disease and received active medication [6–9]; therefore, the differences identified may be related to the medication and the presence of comorbidities in these patients. The results of the changes in the gene expression obtained in these studies may not reflect fully the processes associated with the pathogenesis of PD. Thus, it is necessary to carry out studies in patients at the earliest stages of PD, as this may lead to a better understanding of the processes involved in the development of this disorder.

Because it is very difficult to study endogenous processes that occur directly in the brain of patients with PD during the early symptomatic stages, one of the main approaches that is used to bypass such difficulties is the investigation of changes in gene expression in the fraction of peripheral blood lymphocytes. These cells have several features that are specific to DA neurons, for example, as they produce dopamine receptors and enzymes involved in the synthesis of dopamine [10–13]. Recently, it was shown that several noncoding RNAs change their expression in leucocytes of patients with PD after deep brain stimulation treatment [13, 14]. Furthermore, various studies of changes in gene expression analyses in the peripheral blood of patients with PD have been carried out [15–21]. It was revealed that nine genes (*VDR*,* HIP2*,* CLTB*,* FPRL2*,* CA12*,* CEACAM4*,* ACRV1*,* UTX*, and* SRRM2*) exhibited changed expression in the blood of patients with PD [16, 22]. Although the results of these studies are of great interest, the patients analyzed therein were in advanced stages of PD (stages 2–4 of the Hoehn-Yahr scale), and, in most cases, were receiving drug therapy. To avoid errors in the interpretation of the results of expression profile analyses, it is necessary to compare the data from groups of treated and untreated patients at the early stages of PD (stages 1-2 of the Hoehn-Yahr scale).

To understand better the mechanisms underlying the pathogenesis of PD, we analyzed the expression of 10 genes in the peripheral blood of two groups of patients with PD (i.e., treated and untreated) who were in stages 1-2 of the disease (Hoehn-Yahr scale). The genes that were chosen for this investigation were selected based on an analysis of data published previously. The list included genes involved in the development of monogenic forms of PD (*ATP13A2*,* PARK2*,* PARK7*,* PINK1*,* LRRK2*, and* SNCA*) and genes that exhibited a change in expression that may be associated with the pathogenesis of PD (*ALDH1A1*,* PDHB*,* PPARGC1A*, and* ZNF746*). Previously, it was shown that the expression level of* ALDH1A1* is high in mesencephalic neurons but that the mRNA level of this gene is decreased in patients with PD [23–25].* PDHB* encodes a subunit of pyruvate dehydrogenase and is associated with mitochondrial functioning. It was shown that an increase in the concentration of pyruvate in the plasma is specific to patients with PD [26]. The product of* PPARGC1A*, the PGC-1*α* protein, is a transcriptional activator of mitochondrial biogenesis that, at the same time, protects neurons from oxidative stress by suppressing reactive oxygen species [27, 28].* ZNF746* is highly expressed in the substantia nigra of the brain of mice, and its protein product, PARIS, is involved in mitochondrial biogenesis via the regulation of PGC-1*α* [29].

## 2. Methods

### 2.1. Patients

All patients (Russians residing in the European part of Russia) were diagnosed with PD at the Research Center of Neurology, Russian Academy of Medical Sciences (RAMS), and did not have family history of PD. All patients with PD were selected and studied according to the international Unified Parkinson's Disease Rating Scale (UPDRS) and Hoehn and Yahr scores [30, 31]. The diagnosis of PD was based on the UK PD Brain Bank Criteria [32]. All patients had an early PD stage (stages 1 and 2 of the Hoehn-Yahr scale).

All patients were examined for the presence of frequent mutations in all genes responsible for monogenic forms of PD by using SALSA MLPA P051 Parkinson mix 1 probemix and SALSA MLPA P052 Parkinson mix 2 probemix (MRC-Holland, Netherlands).

In this work, the following groups of patients with PD were studied: (1) untreated patients with the sporadic form of PD (stages 1 and 2 of the Hoehn-Yahr scale) in the moment when blood was taken (35 individuals); (2) treated patients with the sporadic form of PD (stages 1 and 2 of the Hoehn-Yahr scale) (12 individuals). The mean age ± SD at disease onset was 58.6 ± 7.4 years (range: 46–71), and the mean age at enrollment was 59.2 ± 7.1 years (range: 47–73). Patients with PD had no severe concomitant diseases, such as severe cardiovascular disease, cancer, or diabetes mellitus. Treated patients with PD received different medications (dopamine receptor agonists: pramipexole in a dosage of 1.5 mg/day or piribedil in a dosage of 150 mg/day, L-dopa in a dosage of 150–200 mg/day, and amantadine in a dosage of 300 mg/day), either as monotherapy or in various combinations. Forty-four neurologically normal age-matched individuals and 21 patients with different neurological disorders (amyotrophic lateral sclerosis (56.5 ± 2.2), Charcot-Marie-Tooth disease (18.4 ± 3.6), Wilson's disease (22.8 ± 5.2), and cerebellar ataxia (34.4 ± 3.7)) from the same population were studied as controls.

All blood samples were collected with the informed consent of the investigated persons. The study was approved by the Ethics Committee of the Research Center of Neurology.

### 2.2. RNA Isolation

Blood samples were collected from subjects at 8 a.m. before eating and stored at +4°C for no more than 2 h before RNA isolation. Total RNA was isolated from peripheral blood using a ZR Whole-Blood Total RNA Kit (〈〈Zymo Research Corp.〉〉, USA). RNA quantities were determined by spectrophotometry using a Quant-iT RNA BR Assay Kit and a Qubit fluorimeter (Invitrogen, USA). RIN values were measured by using The Agilent 2100 Bioanalyzer system (Agilent Technologies, USA) and were above 8.

### 2.3. Expression Analysis

The gene expression analysis was carried out using the reverse transcription reaction and real-time PCR with TaqMan probes. Single-stranded cDNAs were produced in three replicates using 50 ng of total RNA, a specific reverse primer (Table 1), and the RevertAid H Minus First Strand cDNA Synthesis Kit (Fermentas, Lithuania), according to the manufacturer's recommendations.

The sequences of gene-specific primers and probes are presented in Table 1.

Real-time reactions were performed using an ANK-32 machine (Institute for Analytical Instrumentation, St. Petersburg, Russia) and PCR reagents (Syntol, Moscow, Russia). Thermal cycling was performed as follows: (1) 600 s at 95°C and (2) 45 cycles of 50 s at 95°C and 20 s at 60°C.

For each cDNA, the reaction was replicated thrice.* POLR2F* and* PSMA5* transcripts were used as reference genes to normalize gene expression data [33]. Threshold amplification levels (Cp, crossing point) were determined using the second derivative maximum technique and the ANK-32 for Windows v1.1 software (Institute for Analytical Instrumentation).

### 2.4. Statistical Analysis

The sequences of the primers and probes were designed using Beacon designer 7.0 software (Premier Biosoft International, Palo Alto, CA, USA).

Relative levels of the transcripts in the test groups were calculated as *R* = 2^−ΔΔCp^ [34]. The levels of the transcripts studied in the group of healthy controls were set as 1. Data were analyzed using the nonparametric Mann-Whitney *U* test with “Statistica for Windows 8.0” (StatSoft, Inc. (2007)) and MS Excel 2010 (Microsoft). ROC curves and PCR efficiency and specificity were obtained by using and Real Statistics Resource Pack of MS Excel 2013 (Microsoft).

## 3. Results

An analysis of changes in the mRNA levels of the genes* ALDH1A1*,* ATP13A2*,* LRRK2*,* PARK2*,* PARK7*,* PINK1*,* PDHB*,* PPARGC1A*,* SNCA*, and* ZNF746* was performed in the peripheral blood of treated and untreated patients at the early stages of PD (stages 1-2 of the Hoehn-Yahr scale). These transcripts were also analyzed in the control neurological disease group, which included patients with different neurodegenerative diseases (ALS, Charcot–Marie-Tooth disease, Wilson's disease, and cerebellar ataxia), to assess the specificity of the observed changes of transcript levels in the context of the pathogenesis of PD. During the work, it was shown that the representation of the transcripts of* PARK2* and* PPARGC1A* in human lymphocytes was below the level of detection of the method used in this investigation. The results of the expression analysis of the remaining genes are shown in Table 2.

As it can be seen from Table 2, a statistically significant increase by more than 1.5-fold in the expression of the* ATP13A2*,* PARK7*, and* ZNF746* genes was observed in patients with PD. These changes were specific to PD, because the expression levels of these genes in the neurological control group did not differ from the respective levels of expression in healthy controls. Changes in the levels of transcripts were observed in the groups of untreated and treated patients with PD. It should be noted that the changes in the transcript levels of* ZNF746* were not statistically significant in treated patients; however, the dynamics of changes were similar to those observed in untreated patients with PD.

Furthermore, the analysis showed a statistically significant decrease in the expression levels of* SNCA* in patients with PD, by over fourfold; however, these results are not specific to PD, because a significant downregulation of this gene was also found in the neurological disease control group. The results of the expression analysis of the* PINK1* gene were also interesting. There was no significant change in the expression of this gene in the group of patients with PD; however, a dramatic decrease in its mRNA levels (by more than fourfold) was observed in the neurological disease control group.

## 4. Discussion

To date, a large number of studies of the transcriptome have been conducted in patients with PD at advanced stages of the disease, as well as in the postmortem brain material of patients with PD. In such studies, patients were receiving active medication treatment; therefore, the differences discovered may be associated with the medication and the presence of concomitant diseases in patients [6–9, 15–17]. Because it is impossible to study endogenous processes occurring in the brain of patients with PD in the early symptomatic stages, one of the main approaches is to study the changes in gene expression in the more accessible tissues such as cerebrospinal fluid and peripheral blood [13, 15, 16, 22, 35]. The undeniable advantage of the CSF is that it is in the direct contact with the brain and the spinal cord. Currently, studies are carried out to find potential biomarkers in the cerebrospinal fluid of patients with PD [35]; however, this approach also has significant drawbacks. Thus, obtaining samples of cerebrospinal fluid is very complex procedure and may lead to serious complications. Today, more and more attention is paid to the study of peripheral blood of patients with PD. Moreover, lymphocytes are the most accessible cells for research because they possess several characteristics of DA neurons [10–12]. This fact suggests that the changes observed in the peripheral blood of patients with PD may reflect processes of the DA neurons of the substantia nigra.

It should be noted that only those patients who lacked the most frequent mutations associated with PD were selected for the analysis of the levels of the transcripts of the genes investigated and that the cause of the disease was unknown. Thus, two groups of patients with PD (i.e., with and without treatment) were formed for the study, as well as the neurological disease control group, which included patients with ALS, Wilson's disease, and so forth. The selection of patients for the neurological disease control group was carried out so that the pathogenesis of diseases of the patients included in this group was very different from that of the patients included in the PD group and that it was directly related to the central nervous system and the processes of neurodegeneration.

Nonspecific changes in the levels of the transcripts of two genes were identified in this analysis: a significant decrease in the level of the main transcript of* SNCA* was found both in the group of patients with PD and in the neurological disease control group. It is known that SNCA is a presynaptic protein that is actively involved in the function of different types of neurons [36]. Thus, the changes in* SNCA* levels observed here may be associated with the general patterns of development of pathogenic processes in various neurodegenerative disorders. Furthermore, a statistically significant decrease in the levels of the transcript of* PINK1* was shown for the neurological disease control group. PINK1 is one of the key regulators of mitochondrial biogenesis [37]. For example, a serious malfunction of mitochondria, which may lead to the development of neurodegenerative processes [38], was shown in mice model of ALS by introducing the mutant gene hSOD1. Probably, the change in the mRNA level of* PINK1* observed in our work either was associated with changes in mitochondrial functions, or may have led to their disruption, which in turn may cause the development of neurodegeneration.

The data obtained in the analysis of changes in the levels of the transcripts of the* ATP13A2*,* PARK7*, and* ZNF746* genes were the most interesting. A statistically significant increase in the mRNA levels of these genes was observed in the group of untreated patients with PD but not in the neurological disease control group. These changes were specific, and these genes may play an important role in the pathogenesis of PD, including the early clinical stages of the disease. The elevated levels of the transcripts of these three genes were not related to the therapy of patients because the observed changes in treated patients were similar to those observed in untreated patients with PD.

*PARK7* and* ATP13A2* are genes that are responsible for monogenic forms of PD and are associated with the development of the hereditary form of PD; however, there are no published data on changes in their expression levels in patients with PD. The increase in the mRNA level of* PARK7* may indicate a compensatory phenomenon that develops in response to oxidative stress in cells, as it has been shown that the product of this gene, the DJ-1 protein, is able to protect the cells via autoxidation [39].* ATP13A2* encodes a lysosomal ATPase, and changes in its mRNA levels may indicate disturbances in processes that are associated with the removal of harmful components from cells [40].

However, the greatest changes in expression levels were observed for the* ZNF746* gene in the group of untreated patients with PD. Although the role of this gene in the pathogenesis of PD is not clear, there is evidence of a high level of its protein product, PARIS, in the striatum and substantia nigra [29]. PARIS is a transcriptional repressor of PGC-1*α* and a transcriptional coactivator of the peroxisomal receptor, which is involved in the activation of mitochondrial biogenesis and also undergoes ubiquitination by Parkin. PARIS degradation via Parkin triggers the expression of PGC-1*α* and the subsequent activation of mitochondrial biogenesis [41]. Thus, increased mRNA levels of* ZNF746* can affect the inhibition of mitochondrial biogenesis via the repression of PGC-1*α*, leading to the development of neurodegenerative processes.

## 5. Conclusion

Based on these results, it can be suggested that the upregulation of the mRNA levels of* ATP13A2*,* PARK7*, and* ZNF746* in untreated patients in the earliest clinical stages can also be observed in the preclinical stages of PD and that these genes can be considered as potential biomarkers of the preclinical stage of PD. However, further studies are needed to examine this hypothesis, especially in patients in the preclinical stage of PD.

## Figures and Tables

**Table 1:** Sequences of gene-specific primers and probes.

Gene	Nucleotide sequences	Size of amplicon	*T*_*a*_ (°C)^b^
*ALDH1A1* NM_000689.4^a^	Probe: 5′-ROX-CACTTACCACGCCATAGCAATTCACC-BHQ2-3′ Forward primer: 5′-TAAAGCCATAACAATCTCCTCTG-3′ *Reverse primer: 5*′*-ACATCTTGAATCCACCAAAGG-3*′	101	P^c^: 62.2 F^d^: 54.8 R^e^: 54.4

*ATP13A2* NM_022089.2	Probe: 5′-ROX-AGTCCACCCACTCTTCCTCTCCCC-BHQ2-3′ Forward primer: 5′-TCTAAGGGACATGGTCAAGTTG-3′ *Reverse primer: 5*′*-CAGTCTCCGGGCACTAGC-3*′	99	P: 62.5 F: 56.2 R: 56.9

*LRRK2* NM_198578.3	Probe: 5′-ROX-AAGCAACACTTCCCTGGATATAATGGCA-BHQ2-3′ Forward primer: 5′-TGGCTGTAAAATGCTAAATCATC-3′ *Reverse primer: 5*′*-CGTTTCATAACTGTTAGTATTTTGG-3*′	96	P: 63.6 F: 54.4 R: 54.2

*PARK2* NM_004562.2	Probe: 5′-ROX-TTCACGACCCTCAACTTGGCTACTCCCTG-BHQ2-3′ Forward primer: 5′-CTGTGTGACAAGACTCAATGATCG-3′ *Reverse primer: 5*′*-CCGGTTGTACTGCTCTTCTCC-3*′	142	P: 67.2 F: 57.9 R: 58.4

*PARK7* NM_007262.4	Probe: 5′-ROX-TCCTACTGCTCTGTTGGCTCATGAA-BHQ2-3′ Forward primer: 5′-TGATAGCCGCCATCTGTG-3′ *Reverse primer: 5*′*-CTTTAGCAAGAGGGTGTGTTG-3*′	90	P: 61.7 F: 54.2 R: 55.4

*PDHB* NM_000925.3	Probe: 5′-ROX-ACATCAATTCATTCTCTAGCACCACCACTG-BHQ2-3′ Forward primer: 5′-TGGAATTCAGAGGATGCTAAAGG-3′ *Reverse primer: 5*′*-CGGAGGAAATTCAAAAGGAACC-3*′	111	P: 63.1 F: 56.9 R: 56.4

*PINK1* NM_032409.2	Probe: 5′-ROX-AGCCATCTTGAACACAATGAGCCAGG-BHQ2-3′ Forward primer: 5′-AACATCTCGGCAGGTTCC-3′ *Reverse primer: 5*′*-CTTGGATTTTCTGTAAGTGACTG-3*′	120	P: 63.6 F: 54.4 R: 54.3

*PPARGC1A* NM_013261.3	Probe: 5′-ROX-AAACCAACAACTTTATCTCTTCCTCTGACCC-BHQ2-3′ Forward primer: 5′-CAGTCGCAGTCACAACAC-3′ *Reverse primer: 5*′*-TTCAATAGTCTTGTTCTCAAATGG-3*′	111	P: 62.9 F: 54.9 R: 54.3

*SNCA* NM_000345.3	Probe: 5′-ROX-TGTTCTCTATGTAGGCTCCAA-BHQ2-3′ Forward primer: 5′-AGCAGGAAAGACAAAAGAGG-3′ *Reverse primer: 5*′*-TTGCTCTTTGGTCTTCTCAG-3*′	100	P: 55.7 F: 53.5 R: 53.3

*ZNF746* NM_001163474.1	Probe: 5′-ROX-ACCCCAGTCCAGGCTCGG-BHQ2-3′ Forward primer: 5′-CCTGGCCCCAAGATTCCAG-3′ *Reverse primer: 5*′*-CTGCTTGATCTGCATCAAGAGGTG-3*′	93	P: 60.7 F: 56.5 R: 57.7

*POLR2F* NM_021974.3	Probe:5′-FAM-CTTCATCCTCCTCCACATCATCAAAGTCGTCG-BHQ1-3′ Forward primer: 5′-ATGTCAGACAACGAGGACAATTTTG-3′ *Reverse primer: 5*′*-TCTTCGGCATTCTCCAAGTCATC-3*′	89	P: 66.3 F: 59.4 R: 59.4

*PSMA5* NM_001199772.1	Probe: 5′-FAM-AGCCATCAAGTCTTCACTCATCATCCTC-BHQ1-3′ Forward primer: 5′-AGAAGTTTACCACAAGTCTATGAC-3′ *Reverse primer: 5*′*-CATTCAGCTTCTCCTCCATTAC-3*′	89	P: 64.0 F: 55.3 R: 55.1

^a^Accession numbers in GenBank database; ^b^ *T*_*a*_ (°C): annealing temperature; ^c^P: probe: ^d^F: forward primer; ^e^R: reverse primer. FAM and  ROX: fluorescent dyes; BHQ1 and BHQ2: fluorescent quenchers. Primers in italics are used in the reverse transcription.

**Table 2:** Relative expression levels of the genes investigated.

Gene	Expression level in untreated patients Me^a^ (25%–75%)^b^	Expression level in treated patients Me (25%–75%)	Expression level in neurological control Me (25%–75%)
*ALDH1A1*	1.08 (0.71–1.53)	1.60 (0.94–2.08)	0.95 (0.33–1.64)
*ATP13A2*	**1.94 (1.43–3.03)** ^c^	**2.14 (1.29–4.86)**	0.86 (0.46–1.62)
*LRRK2*	0.44 (0.31–1.26)	0.56 (0.41–1.42)	0.83 (0.37–1.35)
*PARK7*	**1.68 (0.96–2.51)**	**1.60 (1.32–2.20)**	1.00 (0.64–1.50)
*PINK1*	0.75 (0.25–1.40)	1.29 (0.28–3.37)	**0.26 (0.12–0.56)**
*PDHB*	1.46 (0.65–2.16)	2.10 (0.58–2.36)	1.32 (1.01–1.55)
*SNCA*	**0.24 (0.06–1.16)**	**0.16 (0.04–0.24)**	**0.14 (0.02–0.99)**
*ZNF746*	**2.33 (0.96–4.60)**	2.46 (0.66–5.01)	1.07 (0.33–1.64)

^a^Me: median; ^b^25%–75%: 25–75 percentiles. ^c^The data in bold are statistically significant (*p* < 0.05).
